# Domestic Abuse of Codeine: A Case Study of Non-Medical Use Leading to Fatal Outcome

**DOI:** 10.3390/toxics14010071

**Published:** 2026-01-13

**Authors:** Karolina Mrochem, Ewelina Pieprzyca, Gabriela Skalniak, Jakub Obrzut, Julia Cieśla, Elżbieta Chełmecka, Marcin Tomsia, Rafał Skowronek

**Affiliations:** 1 Department of Forensic Medicine and Toxicology, Faculty of Medical Sciences in Katowice, Medical University of Silesia, 18 Medyków Street, 40-752 Katowice, Poland; kmrochem@sum.edu.pl (K.M.);; 2 Faculty of Medical Sciences in Katowice, Medical University of Silesia, 18 Medyków Street, 40-752 Katowice, Poland; 3 Department of Medical Statistics, Faculty of Pharmaceutical Sciences in Sosnowiec, Medical University of Silesia, 41-200 Sosnowiec, Poland

**Keywords:** codeine, cold water extraction, fatal poisoning, forensic toxicology, OTC drugs

## Abstract

Codeine, an opioid analgesic present in many over-the-counter (OTC) formulations, is frequently misused through non-medical extraction techniques such as cold water extraction (CWE). These practices carry substantial risks, including incomplete removal of hepatotoxic co-formulants, contamination, and highly unpredictable dosing. We report a fatal case of a 29-year-old man who ingested codeine extracted from Antidol^®^ tablets in combination with energy drinks and psychotropic medications. Post-mortem LC–MS/MS analysis revealed the presence of codeine (0.66 µg/mL), morphine (0.02 µg/mL), hydroxyzine (2.52 µg/mL), alprazolam (0.15 µg/mL), paracetamol (30.64 µg/mL), and additional substances in blood samples. Concentrations of codeine and hydroxyzine exceeded therapeutic ranges and were consistent with values reported in fatal intoxications, confirming a poly-drug poisoning. This case highlights the danger associated with non-medical codeine use, particularly when combined with central nervous system (CNS) depressants, and underscores the need for stricter regulation of OTC codeine-containing products as well as improved public awareness of the risks associated with domestic extraction methods.

## 1. Introduction

**Figure 1:**
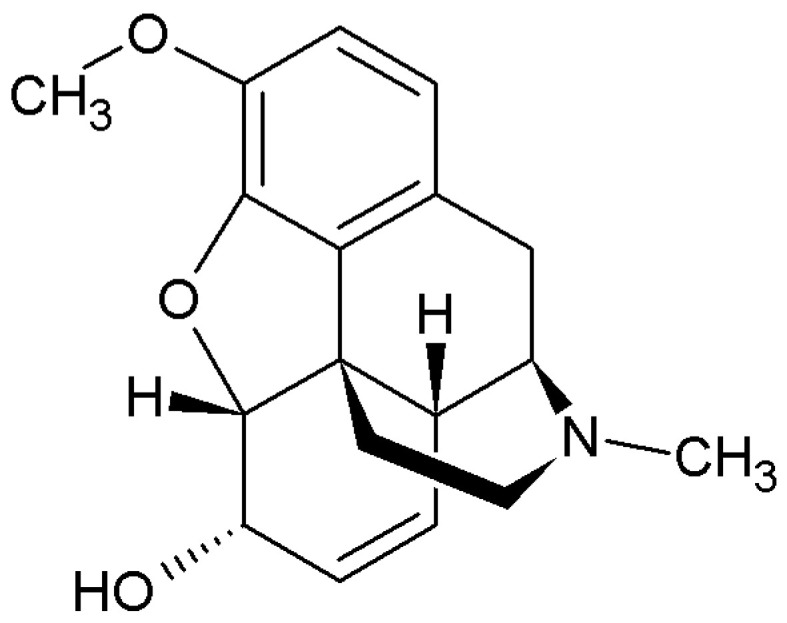
Codeine molecule (created with ChemSketch 12.0).

Codeine (Figure 1) (Latin: *Codineum*, also known as methylmorphine or morphine methyl ether, C_18_H_21_NO_3_) is a weak opioid analgesic and a morphine derivative widely used for its analgesic and antitussive properties [1,2,3]. It is a natural alkaloid of *Papaver somniferum* and a common component of both prescription and over-the-counter (OTC) pharmaceutical preparations, including Antidol^®^, Solpadeine^®^, and Thiocodin^®^ [2,4]. Despite ongoing debate regarding its clinical effectiveness, codeine remains listed in the World Health Organization’s Model List of Essential Medicines [5,6,7].

The pharmacological and toxicological effects of codeine are largely determined by hepatic metabolism. Codeine is primarily biotransformed into morphine via the cytochrome P450 isoenzyme CYP2D6, which governs both its analgesic efficacy and toxic potential [8]. Interindividual variability in CYP2D6 activity markedly influences therapeutic response and the risk of toxicity [9].

Many OTC formulations combine codeine with non-opioid analgesics, including paracetamol, ibuprofen, and acetylsalicylic acid. Misuse, chronic consumption, or overdose of these combination products may result in severe and potentially fatal intoxication, with toxicity extending beyond opioid effects to include hepatotoxicity, nephrotoxicity, and gastrointestinal bleeding related to the co-formulated substances [10].

## 2. Pharmacokinetics and Metabolism

Codeine is a widely used opioid with antitussive, analgesic, and antidiarrheal properties. Therapeutic doses range from 30 to 60 mg every four hours, with a maximum daily dose of 240 mg [10,11]; low doses (10–20 mg) primarily exert antitussive effects, while higher doses provide moderate analgesia [4]. Compared to morphine, codeine has substantially weaker analgesic and narcotic effects [11].

Non-medical use of codeine may induce euphoria, drowsiness, emotional detachment, or appetite suppression; however, overdose may lead to nausea, vomiting, dizziness, constipation, and in severe cases, profound sedation, respiratory depression, hypotension, coma, or death [11,12,13]. These effects largely reflect the metabolic conversion of codeine to morphine, a more potent opioid.

Codeine is a prodrug with low affinity for μ-opioid receptors and requires biotransformation to morphine to exert its pharmacological effects. It is rapidly metabolized, with peak plasma concentrations of metabolites reached within approximately 20 min [14,15]. The predominant metabolic pathway is glucuronidation to inactive codeine-6-glucuronide; approximately 10% undergoes O-demethylation to morphine or N-demethylation to norcodeine [16].

Interindividual variability in codeine metabolism may substantially influence both therapeutic response and toxic effects. Differences in metabolic capacity, whether due to genetic factors or drug–drug interactions, contribute to unpredictable clinical outcomes and complicate the interpretation of toxicological findings in forensic cases [16,17,18,19,20]. Consequently, codeine toxicity cannot be reliably inferred from the ingested dose alone, particularly in post-mortem investigation.

## 3. Analytical Detection Methods

A wide range of analytical methods have been developed for the detection of codeine in biological and non-biological matrices. In forensic and clinical toxicology, blood and urine are the most commonly analyzed specimens, although alternative matrices such as saliva or dental tissue have also been investigated [21]. The choice of analytical technique depends on the required sensitivity and specificity, the type of biological material, and the available instrumentation.

Commonly applied methods include immunochemical screening techniques, gas chromatography-mass spectrometry (GC-MS) [21,22], liquid chromatography-tandem mass spectrometry (LC-MS/MS) [14,20,22], immunochromatographic assays, thin-layer chromatography, spectrophotometry, spectrofluorometry, and chemiluminescence [23].

## 4. Regulatory Landscape and Public Health Impact

Codeine misuse and dependence have been consistently documented over several decades [24,25,26]. In response, national and international regulatory authorities have implemented heterogeneous policies governing the sale and distribution of codeine-containing medicinal products. The regulatory status of codeine—particularly its availability as an over-the-counter (OTC) medication—varies substantially between countries and has been shown to influence the incidence of codeine-related poisonings and fatalities. Table 1 summarizes the regulatory landscape for codeine-containing products in selected countries.

In countries such as Japan, the United Kingdom, Denmark, and Poland, selected codeine-containing formulations remain available without prescription, typically subject to restrictions on pack size, dose, or formulation. In contrast, Australia, Germany, and France have adopted more restrictive approaches, classifying all codeine-containing medicinal products as prescription-only. These divergent regulatory strategies allow assessment of the public health impact of codeine control measures. Notably, in Australia, reclassification of codeine to prescription-only status was followed by a significant reduction in codeine-related poisonings and mortality, supporting the effectiveness of stricter regulatory control [27,28].

Owing to the widespread availability of OTC codeine-containing medications, community pharmacists are often among the first healthcare professionals to identify potential patterns of misuse. Studies conducted among pharmacy staff in Poland indicate that pharmacists, including those with relatively limited professional experience (2–5 years), are capable of recognizing behaviors suggestive of non-medical drug use [29,30]. Common indicators include repeated purchases of large quantities, acquisition of multiple packages during a single visit, and purchases occurring during evenings or weekends. Observable behavioral or physical signs, such as marked fatigue or atypical conduct, may further raise suspicion of substance misuse.

A survey conducted in a Polish province in 2014–2015 examined misuse of OTC medications, including codeine-containing products. Among pharmacists who identified behaviors suggestive of misuse, 38.2% reported attempting to prevent the transaction by declaring the product unavailable, whereas 34.0% dispensed the medication despite their concerns, citing the ease of obtaining it elsewhere. Additionally, 21.9% reported dispensing the product after receiving a satisfactory explanation from the customer, while 5.9% acknowledged doing so due to fear of the customer’s reaction to refusal [29].

In 2016, the Polish Minister of Health introduced regulations limiting codeine sales to a maximum of 240 mg per preparation per transaction [31]. However, the absence of a mechanism restricting the purchase of multiple packages in separate transactions allows circumvention of existing limits, potentially facilitating access to larger quantities of codeine, with implications for public health and clinical toxicology.

## 5. Non-Medical Use and Extraction Methods

Codeine is frequently used for non-medical purposes, most commonly to induce euphoria or a sense of well-being, and less frequently, hallucinatory-like effects [27]. Online forums and social media platforms facilitate the dissemination of experiences and guidance related to recreational use and extraction from pharmaceutical preparations [32]. Various forms of misuse have been described, including the consumption of “purple drank”, a mixture of codeine-containing cough syrup with alcoholic or non-alcoholic beverages, primarily reported in the United States [33,34]. Another highly potent derivative, desomorphine (“Krokodil”), obtained through chemical modification of codeine, has been associated with severe abuse-related harm, particularly in Russia, Ukraine, and neighboring regions [27].

A major public health concern is the misuse of combination products containing codeine and non-opioid analgesics, such as paracetamol (e.g., Antidol^®^, Solpadeine^®^), ibuprofen (e.g., Nurofen Plus^®^), or sulfogaiacol (e.g., Thiocodin^®^). Because high doses of paracetamol are associated with severe and potentially fatal hepatotoxicity, users often attempt to separate codeine from these formulations.

The most commonly reported method is cold water extraction (CWE), which exploits the differences in solubility between codeine salts and paracetamol [35]. Although intended to reduce paracetamol content, CWE is inherently unreliable; incomplete filtration or improper technique may result in substantial residual paracetamol, posing a significant risk of fatal poisoning [22,36].

More complex extraction methods involving strong bases and organic solvents have also been described [37,38]. While these approaches may reduce contamination with co-formulated analgesics, they introduce additional hazards related to solvent toxicity and corrosive reagents, further increasing the risk of severe or fatal outcomes.

Importantly, codeine dependence may also develop unintentionally, particularly among individuals who underestimate its addictive potential or associated health risks. Limited awareness of these risks—sometimes reinforced by the perception of codeine as a relatively “safe” OTC medication—contributes to the persistence of codeine-related substance use disorder [7].

## 6. Case Report: Fatal Codeine Intoxication

A 29-year-old man with a documented history of anxiety-related depressive disorder was found unresponsive in bed at his residence. Resuscitation attempts by emergency medical services were unsuccessful, and death was pronounced at the scene. Vomitus was observed near the head of the deceased.

**Figure 2:**
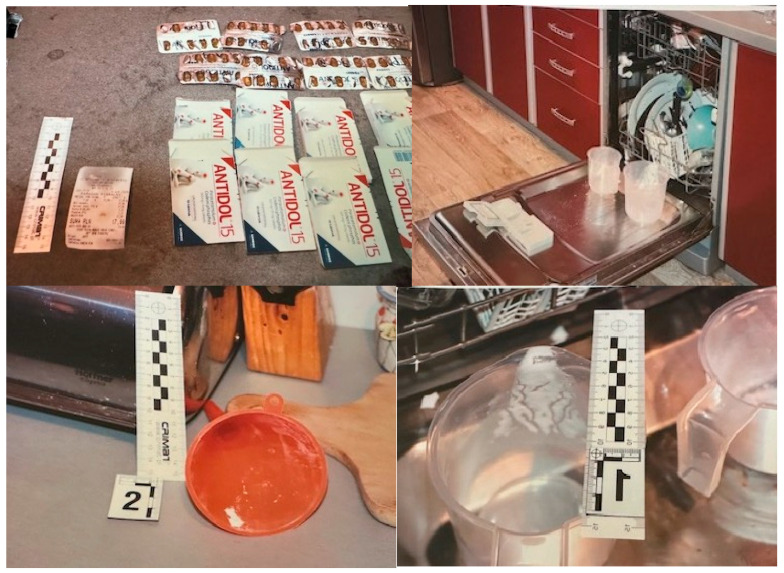
Evidence found on-site, indicating home-made production.

According to information provided by his partner, the decedent had a history of non-medical use of codeine extracted from OTC medications. He reportedly used extracted codeine recreationally for approximately four months, with a frequency of about once per month. On the day of death, he allegedly purchased and consumed the extracted contents of eight packages of Antidol^®^, a codeine-paracetamol combination product, together with unspecified quantities of energy drinks (available only to adults in Poland). The extraction was reportedly performed using a homemade heating-and-cooling method (Figure 2).

Scene investigation revealed multiple empty blister packs of Antidol^®^, containers of energy drinks, and blister remnants of alprazolam and hydroxyzine, as well as other OTC medications. Given the presence of multiple psychoactive substances, a medicolegal autopsy and comprehensive toxicological analysis were conducted.

### 6.1. Medicolegal Autopsy

Body fluids sampling was approved by the Bioethical Commission of the Medical University of Silesia in Katowice (decision no. KNW/0022/KB/207/19, approval date: 14 October 2019). Femoral blood and urine samples were collected during a medico-legal autopsy commissioned by the Prosecutor’s Office.

The autopsy was performed to determine the cause and manner of death. External examination and internal dissection revealed non-specific findings, including cerebral edema and congestion of internal organs, consistent with acute circulatory failure. No traumatic injuries were identified, and no anatomical abnormalities sufficient to explain the cause of death were observed at the macroscopic level. Histopathological examination was not ordered by the prosecutor.

### 6.2. Toxicological Analysis

Blood and urine samples were collected during the autopsy in accordance with the recommendations of the Polish Society of Forensic Medicine and Criminology for comprehensive toxicological analysis. Initial screening was performed using enzyme-linked immunosorbent assays (ELISA), yielding positive results for both benzodiazepines and opioid alkaloids. The presence of ethanol was excluded using headspace gas chromatography–mass spectrometry (HS–GC–MS).

Confirmatory and quantitative analyses were conducted using liquid chromatography–triple quadrupole tandem mass spectrometry (LC–MS/MS) on Thermo Scientific (Waltham, MA, USA) TSQ Quantum Access MAX system. Sample preparation involved a liquid–liquid extraction (LLE) procedure: protein precipitation with acetonitrile, followed by extraction with ethyl acetate at pH 9.0 using Tris buffer. The extracts were evaporated to dryness and reconstituted in methanol (MeOH) prior to instrumental analysis.

Chromatographic separation was performed on a Thermo Scientific C18 column (150 × 2.1 mm ID, 5 μm). The mobile phase consisted of two components: phase A, comprising water with 0.2% formic acid and 0.002 M ammonium formate, and phase B, consisting of acetonitrile with 0.2% formic acid and 0.002 M ammonium formate.

The screening method routinely employed in the Department of Forensic Medicine and Forensic Toxicology in Katowice has been validated according to SWGTOX guidelines and encompasses several hundred analytes, including narcotics, psychoactive substances, and pharmaceuticals relevant to the present [39]. This approach enabled qualitative and quantitative assessment of multiple compounds in blood and urine samples, as summarized in Table 2. Due to the low blood concentration of morphine, additional targeted analyses for morphine and codeine were performed to ensure accurate quantification. Further methodological details are provided in the Supplementary Material.

The toxicological findings identified codeine, alprazolam, and hydroxyzine as the primary substances of concern. The blood concentration of codeine (0.66 μg/mL) exceeded the therapeutic range (0.03–0.47 μg/mL), fell within the toxic range (0.5–1 μg/mL), and was consistent with levels reported in fatal cases (0.45–48 μg/mL) [40,41,42]. Similarly, hydroxyzine was present at 2.52 μg/mL, above the toxic threshold and within the range observed in fatal intoxications.

Morphine was detected at 0.02 μg/mL, confirming metabolic conversion of codeine to its active form and contributing to overall toxicity. Importantly, 6-monoacetylomorphine, a specific marker of heroin use, was absent, supporting the interpretation that morphine originated from codeine metabolism rather than heroin exposure [43]. The concomitant use of these three central nervous system depressants is particularly concerning, as their synergistic effects on CNS and respiratory function would have been markedly amplified, ultimately leading to a fatal outcome.

## 7. Discussion

The fatal outcome in this case was most plausibly associated with the synergistic depressant effects of multiple CNS-active substances, including codeine, hydroxyzine, and alprazolam. While each of these compounds exerts CNS depressant effects independently, their concurrent use markedly increases toxicodynamic risk. Codeine, via its active metabolite morphine, induces sedation and respiratory depression through μ-opioid receptor activation. Hydroxyzine, a sedating antihistamine, further suppresses central nervous activity, while its metabolite cetirizine may contribute additional sedative burden. Alprazolam, a benzodiazepine, enhances GABAergic neurotransmission, leading to profound sedation, anxiolysis, and muscle relaxation. The combined presence of these substances likely produced additive and synergistic effects, particularly on respiratory drive, substantially increasing the risk of hypoventilation, hypoxia, and cardiovascular compromise, even when individual substances may not have reached independently lethal concentrations.

Other detected substances may have modulated the overall toxicological profile. Paracetamol, present as a co-formulated component, could have contributed to systemic stress, while caffeine ingested via energy drinks may have transiently counteracted early sedation, facilitating continued intake of depressant substances and delaying overdose recognition. Venlafaxine and lamotrigine, although detected at non-toxic levels, may have exerted minor additive effects on neuronal excitability and central neurotransmission.

This case also illustrates considerable interindividual variability in codeine metabolism, the impact of drug–drug interactions, and the challenges in accurately reconstructing the ingested dose. Codeine is metabolized to morphine primarily via CYP2D6, whereas CYP3A4 mediates conversion to norcodeine. Genetic polymorphisms or the presence of CYP2D6 inhibitors can significantly affect the extent of morphine formation, thereby modifying both therapeutic and toxic effects. Although CYP2D6 genotyping was not feasible in this case, such variability may have influenced the observed toxic outcome. It should be noted that CYP2D6 genotyping is rarely feasible in forensic cases due to legal, ethical, and practical constraints; thus, variability in metabolic capacity must often be inferred from toxicological findings, pharmacokinetic knowledge, and circumstantial evidence rather than directly confirmed.

Variability in post-mortem codeine and morphine concentrations, as well as in their relative ratios, has been previously reported in fatal opioid intoxications, illustrating the limitations of using the codeine/morphine ratio as a sole interpretative parameter [43]. Reliable dose estimation in post-mortem investigations is inherently limited by factors such as unknown timing of ingestion, variable absorption and metabolism, post-mortem redistribution, and the use of non-medical extraction methods.

The presented case is not an isolated case. Compared with previously reported cases, such as Fais et al., where alcohol was the only additional psychoactive substance [22], the present case involved multiple CNS depressants, highlighting that fatal outcomes may occur both in isolation and in combination with other depressants. Population-level analyses further underscore the public health significance of codeine-related mortality, with trends in accidental and intentional overdoses emphasizing the need for comprehensive preventive strategies [44].

These observations highlight the importance of a multidisciplinary approach in forensic medicine, integrating toxicology, histopathology, clinical pharmacology, and legal context to achieve robust and defensible medico-legal conclusions. Such an approach is essential for the accurate interpretation of complex fatal intoxications involving poly-drug exposure and interacting pharmacological mechanisms.

## 8. Conclusions

This case report highlights the critical public health issue posed by the non-medical use of over-the-counter (OTC) codeine-containing products. The high accessibility of these drugs, combined with insufficient awareness of the risks associated with domestic extraction methods and poly-drug use, creates a significant potential for fatal intoxication. The tragic death of the 29-year-old man underscores that even a single episode of such misuse can have catastrophic consequences.

Documented cases of fatal codeine poisoning, including this one, confirm the scale of the problem and the urgent need for enhanced public health interventions. More stringent regulation of OTC codeine-containing products, together with targeted public awareness campaigns addressing both codeine misuse and domestic extraction practices, remains essential.

Although histopathological examination could have provided additional information on organ-level alterations, the absence of such data does not preclude a reliable forensic assessment. In this case, the comprehensive toxicological findings, scene investigation, circumstantial evidence, medical history, and well-documented synergistic effects of CNS depressants provided a sufficient basis to support the proposed mechanism of death.

## Figures and Tables

**Table 1:** Summary of codeine-containing medication regulations in selected countries.

Country	Availability of Codeine Drugs as OTC	Max. Level Allowed in OTC	Dosage Units Sales per 1000 Population (2013–2019)	Regulations of Codeine OTC Drugs Sales		
				Must Be Sold Under Supervision of Pharmacist	Limits on Quantity of Tablets Sold in a Single Transaction	Age Restriction Condition of Sale
Australia	No	None	Unknown	-	-	-
Bulgaria	Yes	30 mg	12,268 ± 8536	Yes	No	No
Denmark	Yes	9.6 mg	Unknown	No	Yes	Yes
France	No (since 2017)	None	19,895 ± 13,365 ^2^	-	-	-
Germany	No (since 2018)	None	0.895 ± 0.2	-	-	-
Japan	Yes	-	12,268 ± 8536 ^1^	Yes	Yes	Yes
Poland	Yes	15 mg	9228 ± 797	Yes	Yes	No
United Kingdom	Yes	12.8 mg	17,164 ± 497	Yes	Yes	Yes

^1^ sales data 2015–2019. ^2^ sales data 2013–July 2017.

**Table 2:** Concentrations of detected substances in blood and urine samples collected post-mortem.

Detected Substance	Blood Concentration (µg/mL)	Urine Concentration (µg/mL)
codeine (free)	0.66 ^1,2^	11.99
morphine (free)	0.02	1.45
paracetamol	30.64 ^1^	58.00
caffeine	1.31	3.03
hydroxyzine	2.52 ^1,2^	10.73
cetirizine	1.77	35.32
alprazolam	0.15	0.18
venlafaxine	0.23	1.87
lamotrigine	5.62	20.23

^1^ concentration within the range reported as toxic [40,41,42], ^2^ concentration within the range reported as fatal [40,41,42].

## Data Availability

The data presented in this study are available on request from the corresponding author.

## Supplementary Materials

The following supporting information can be downloaded at: https://www.mdpi.com/article/10.3390/toxics14010071/s1, Supplementary materials.

## Abbreviations

The following abbreviations are used in this manuscript:

OTC	Over-the-counter
CWE	Cold Water Extraction
LC-MS/MS	Liquid Chromatography-Tandem Mass Spectrometry
CNS	Central Nervous System
WHO	World Health Organization
GC-MS	Gas Chromatography-Mass Spectrometry
KOH	Potassium hydroxide
NaOH	Sodium hydroxide
ELISA	Enzyme-Linked Immunosorbent Assay
LLE	Liquid–Liquid Extraction
Tris	Tris(hydroxymethyl)aminomethane
C18	Octadecylsilane
ID	Internal Diameter
SRM	Selected Reaction Monitoring
ACN	Acetonitrile
MeOH	Methanol
*v*/*v*	volume/volume
