# Supplementary material for: Domestic Abuse of Codeine: A Case Study of Non-Medical Use Leading to Fatal Outcome
Source: Toxics. 2026 Jan 13;14(1):71. doi: 10.3390/toxics14010071 (PMC12846103; doi:10.3390/toxics14010071)
Supplement: Supplementary file 1 [file toxics-14-00071-s001.zip › toxics-4050616-supplementary.pdf]

Supplementary materials

Blood and urine sampling

Blood and urine samples were collected during the autopsy in accordance with the recommendations of the Polish Society of Forensic Medicine and Criminology for comprehensive toxicological analysis. Both matrices were stored at 4°C until analysis.

Chemicals

Certified reference materials were obtained from various sources: LGC Standards GmbH (Wesel, Germany), Lipomed AG (Arlesheim, Switzerland), and Merck (Darmstadt, Germany). All chemicals and reagents used for the analyses were of the highest commercially available purity. Acetonitrile, ethyl acetate, formic acid, t-butanol, methanol, tris(hydroxymethyl)aminomethane (TRIS) hydrochloride, 35% ammonium formate, and water LiChrosolv® were purchased from Merck (Darmstadt, Germany). The immunoenzymatic ELISA Forensic Drug Test kits were purchased from Neogen (Lansing, Michigan, USA).

Preparation of biological specimens

Biological samples were prepared using a liquid–liquid extraction (LLE) procedure. To 2 mL of each body fluid, 2 mL of acetonitrile, 2 mL of TRIS buffer (pH 9), and 20 mL of ethyl acetate were introduced. The mixtures were shaken for 3 hours and then centrifuged for 15 minutes. The resulting supernatants were collected and evaporated to dryness under a gentle stream of nitrogen at room temperature. The dry residues were reconstituted in 1 mL of the mobile phase and transferred to glass inserts in vials for subsequent analysis by liquid chromatography–mass spectrometry (LC–MS/MS).

Analytical methods

Extracts were analyzed on two independent chromatographic–mass spectrometric platforms: HPLC-MS Analysis Instrument:

- Analyses were performed on a Thermo Scientific TSQ Quantum Access Max mass spectrometer (Thermo Scientific, Waltham, USA). Separation was performed on a Thermo Scientific C18, 150 × 2.1 mm ID, particle 5 µm column, thermostated at 25 °C. Phase A was water, which contained 0.2% formic acid and 0.002 M of ammonium formate, and phase B was acetonitrile with 0.2% formic acid and 0.002 M of ammonium formate. Gradient program: 5% B (0–2 min) → 70% B (2–35 min)→ 100% B (35-40 min)→ 2 min isocratic; re-equilibration for 10 min. Flow rate: 0.4 ml/min, injection volume: 10 µl, ionization: positive electrospray ionization (ESI+), capillary voltage 2.5 kV, source temperature 325°C, gas flow (nitrogen) 10 L/min, acquisition mode: full scan (m/z 50–650) and MS/MS product ion scans for confirmation.
- UHPLC-MS/MS analyses were performed on a Thermo Scientific TSQ Quantum Access Max mass spectrometer (Thermo Scientific, Waltham, USA). Separation was performed on a Thermo Scientific C18, 150 × 2.1 mm ID, particle 5 µm column, thermostated at 25 °C. Phase A was water, which contained 0.2% formic acid and 0.002 M of ammonium formate, and phase B was acetonitrile with 0.2% formic acid and 0.002 M of ammonium formate. Gradient program (shown in relation to phase B content): 0 min – 5%, 5 min – 70%, 13 min – 100%, 18 min 5%. The mass spectrometer was operated in the multiple reaction monitoring (MRM) mode with transitions at 300.1 → 58.1, 300.1

→ 153.0, and 300.1 → 165.2 for codeine and at 286.0 → 153.1, 286.0 → 165.1, and 286.0 → 181.1 for morphine. The optimized collision energies were 39 eV for transitions at m/z 300.1/58.1, 50 eV for transitions at m/z 300.1/153.0 and 41 eV for transitions at m/z 300.1/165.2 (codeine) and 41 eV for transitions at m/z 286.0/153.1, 37 eV for transitions at m/z 286.0/165.1 and 35 eV for transitions at m/z 286.0/181.1 (morphine). The mass detector parameters were as follows: capillary voltage 2500 V; gas flow (nitrogen) 10 L/min; gas temperature 325 °C; and nebulizer pressure 40 psi.

Thus, the two techniques were complementary: the full-scan method provided broad, non-targeted confirmation and the ability to detect unexpected compounds, while the MRM-based method ensured high sensitivity and specificity for the quantitative determination of the identified analytes. The chromatographic methods are routinely used in our laboratory and have been validated in accordance with SWGTOX guidelines. They cover several hundred substances, including narcotics, psychotropic drugs, and new psychoactive substances (NPSs), as well as a wide range of pharmaceuticals—notably opioids, analgesics, antidepressants, and antipsychotics. Results were processed using Xcalibur v. 2.0 software. The minimum detectable level (LOD) and limit of quantitation (LOQ) for morphine were 10 and 30 ng/mL, respectively, whereas the LOD and LOQ for codeine were 5 and 15 ng/mL, respectively.
